# Supplementary material for: Urinary polycyclic aromatic hydrocarbon metabolites and mortality in the United States: A prospective analysis
Source: PLoS One. 2021 Jun 4;16(6):e0252719. doi: 10.1371/journal.pone.0252719 (PMC8177506; doi:10.1371/journal.pone.0252719)
Supplement: S1 Fig — (DOCX) [file pone.0252719.s001.docx]

S1 Fig. Schematic diagram of exclusion criteria for study population (NHANES 2001-2015).
