# Supplementary material for: Urinary polycyclic aromatic hydrocarbon metabolites and mortality in the United States: A prospective analysis
Source: PLoS One. 2021 Jun 4;16(6):e0252719. doi: 10.1371/journal.pone.0252719 (PMC8177506; doi:10.1371/journal.pone.0252719)

S2 Fig. Directed acyclic graph to determine the relationships between hydroxylated PAH metabolites (OH-PAHs) and mortality.


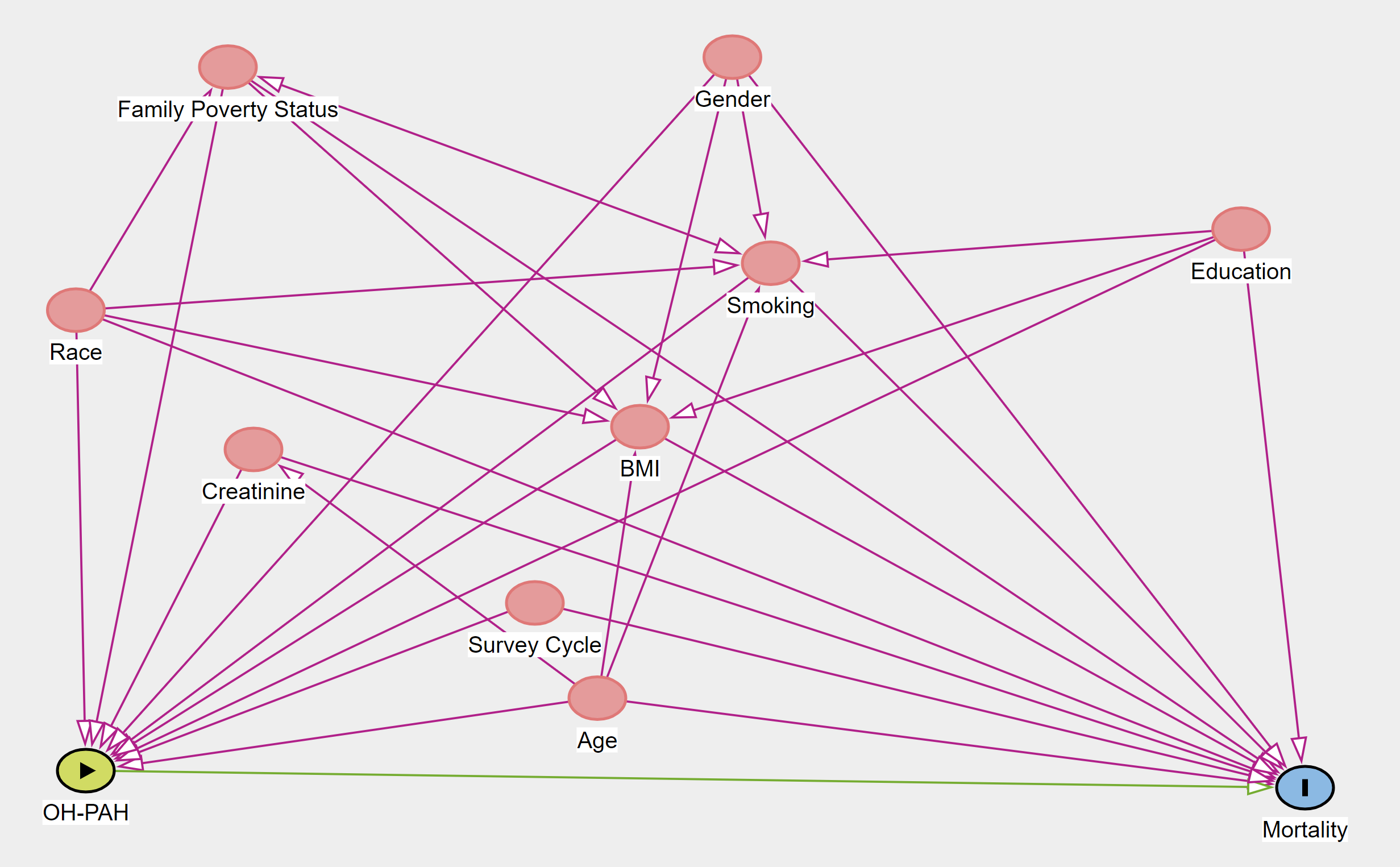

Supplement: S2 Fig — (DOCX) [file pone.0252719.s002.docx]
