# Supplementary material for: Urinary polycyclic aromatic hydrocarbon metabolites and mortality in the United States: A prospective analysis
Source: PLoS One. 2021 Jun 4;16(6):e0252719. doi: 10.1371/journal.pone.0252719 (PMC8177506; doi:10.1371/journal.pone.0252719)

S3 Fig. Weights for urinary OH-PAHs from quantile g-computation, by mortality endpoint.

S3a Fig. Negative and positive weights for OH-PAHs associated with all-cause mortality


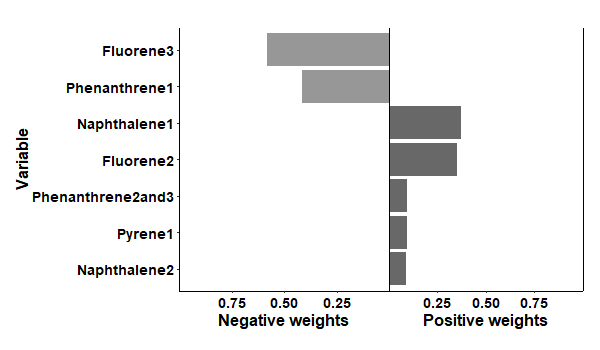


S3b Fig. Negative and positive weights for OH-PAHs associated with cancer-specific mortality


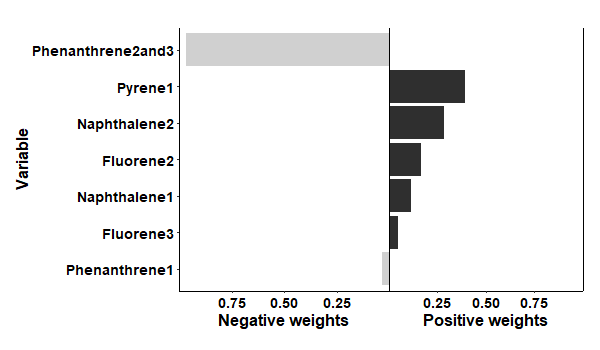


S3c Fig. Negative and positive weights for OH-PAHs associated with cardiovascular-specific mortality


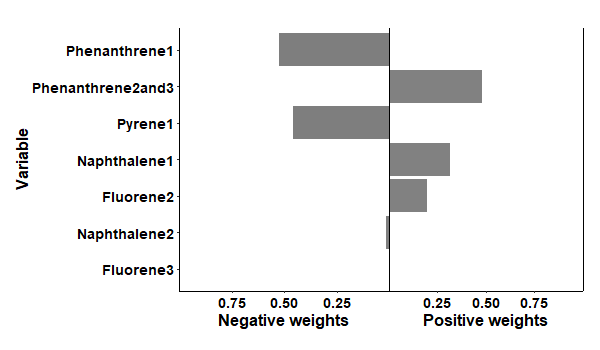

Supplement: S3 Fig — a) Negative and positive weights for OH-PAHs associated with all-cause mortality. b) Negative and positive weights for OH-PAHs associated with cancer-specific mortality. c) Negative and positive weights for OH-PAHs associated with cardiovascular-specific mortality. (DOCX) [file pone.0252719.s003.docx]
