# Supplementary material for: Urinary polycyclic aromatic hydrocarbon metabolites and mortality in the United States: A prospective analysis
Source: PLoS One. 2021 Jun 4;16(6):e0252719. doi: 10.1371/journal.pone.0252719 (PMC8177506; doi:10.1371/journal.pone.0252719)
Supplement: S1 Table — (DOCX) [file pone.0252719.s004.docx]

S1 Table. Baseline descriptive characteristics in NHANES 2001-2014 by mortality status, prior to exclusion criteria and in the final study population.

|  |  |  | **Before exclusion criteria** | |  | **Final study population** | |
| --- | --- | --- | --- | --- | --- | --- | --- |
|  |  |  | **Alive (N=10,888)** | **Deceased (N=1,407)** |  | **Alive (N=8,805)** | **Deceased (N=934)** |
|  | | >LOD (%) | GM (GSE) | | >LOD (%) | GM (GSE) | |
| Urinary OH-PAH (ng/L) | |  |  |  |  |  |  |
|  | 1-naphthalene | 99.85% | 2294.93 (59.52) | 3268.71 (175.80) | 99.85 | 2287.34 (61.24) | 3560.92 (258.57) |
|  | 2-naphthalene | 99.95% | 3756.92 (83.61) | 3134.01 (145.51) | 99.97 | 3759.81 (87.54) | 3126.28 (172.27) |
|  | 3-hydroxyfluorene | 98.39% | 111.48 (2.71) | 108.59 (6.02) | 98.44 | 112.68 (2.92) | 110.58 (7.72) |
|  | 2-hydroxyfluorene | 99.97% | 276.28 (6.22) | 296.28 (14.08) | 99.97 | 277.46 (6.64) | 298.24 (18.11) |
|  | 1-hydroxyphenanthrene | 99.63% | 135.18 (2.20) | 135.97 (4.36) | 99.67 | 135.78 (2.24) | 136.75 (5.55) |
|  | ∑2- & 3-hydroxyphenanthrene | 100% | 150.82 (2.54) | 164.93 (6.74) | 99.82 | 151.00 (2.61) | 163.57 (8.20) |
|  | 1-hydroxypyrene | 61.06% | 114.91 (1.61) | 90.03 (2.44) | 60.95 | 115.05 (1.75) | 89.69 (3.13) |
|  | ∑OH-PAHs^a^ | --- | 8233.83 (180.12) | 8890.81 (417.41) | -- | 8192.57 (187.10) | 9134.58 (534.17) |
|  |  |  |  |  |  |  |  |
| Age at baseline (years) | |  | 44.99 (0.24) | 66.54 (0.55) |  | 45.03 (0.25) | 66.01 (0.65) |
| Follow up time (years) | |  | 7.62 | 5.39 |  | 7.56 | 5.63 |
| Urinary creatinine (g/L) | |  | 0.97 (0.01) | 0.90 (0.02) |  | 1.22 (0.01) | 1.12 (0.03) |
|  | |  |  |  |  |  |  |
|  | |  | Frequency (SE) | |  | Frequency (SE) | |
| Gender | |  |  |  |  |  |  |
|  | Male |  | 47.68 (0.53) | 52.33 (1.64) |  | 48.49 (0.58) | 54.40 (2.11) |
|  | Female |  | 52.32 (0.53) | 47.67 (1.64) |  | 51.51 (0.58) | 45.60 (2.11) |
| Race/ethnicity | |  |  |  |  |  |  |
|  | Non-Hispanic white |  | 68.85 (1.27) | 76.12 (2.22) |  | 69.74 (1.33) | 77.61 (2.00) |
|  | Non-Hispanic black |  | 11.40 (0.70) | 10.21 (1.05) |  | 11.02 (0.70) | 10.52 (1.04) |
|  | Hispanic |  | 13.32 (0.94) | 10.89 (1.99) |  | 12.97 (0.98) | 9.23 (1.47) |
|  | Other |  | 6.43 (0.37) | 2.78 (0.56) |  | 6.28 (0.42) | 2.64 (0.61) |
| Educational attainment | |  |  |  |  |  |  |
|  | Less than high school graduate |  | 16.90 (0.68) | 30.89 (1.67) |  | 16.44 (0.72) | 30.19 (1.78) |
|  | High school graduate |  | 23.21 (0.63) | 26.75 (1.22) |  | 22.82 (0.66) | 29.57 (1.36) |
|  | Some college or above |  | 59.89 (1.00) | 42.36 (1.98) |  | 60.74 (1.03) | 40.24 (1.79) |
| Smoking status^b^ | | | | |  |  |  |
|  | Not active smoker or ETS exposed |  | 71.54 (0.68) | 71.39 (1.49) |  | 71.79 (0.72) | 72.27 (1.72) |
|  | Active smoker or ETS exposed |  | 28.46 (0.68) | 28.61 (1.49) |  | 28.21 (0.72) | 27.74 (1.72) |
| Body mass index (kg/m^2^) | |  |  |  |  |  |  |
|  | Underweight (<18.5) |  | 1.59 (0.15) | 2.34 (0.60) |  | 1.55 (0.17) | 2.67 (0.78) |
|  | Normal (18.5 - 24.9) |  | 31.00 (0.65) | 29.10 (1.75) |  | 30.59 (0.69) | 29.53 (1.73) |
|  | Overweight (25 - 29.9) |  | 33.18 (0.69) | 35.09 (1.79) |  | 33.36 (0.78) | 33.32 (1.64) |
|  | Obese (≥30) |  | 34.22 (0.65) | 33.48 (1.95) |  | 34.50 (0.67) | 34.49 (1.90) |
| Family poverty status^c^ | |  |  |  |  |  |  |
|  | Above the poverty threshold |  | 85.93 (0.59) | 81.81 (1.53) |  | 86.15 (0.57) | 82.17 (1.45) |
|  | At or below the poverty threshold |  | 14.07 (0.59) | 18.19 (1.53) |  | 13.85 (0.57) | 17.83 (1.45) |

Abbreviations: ETS = environmental tobacco smoke; GM = geometric mean; GSE = standard error of the GM; LOD = limit of detection; SE = standard error.

* 21 (out of 12,316 individuals) had ineligible mortality status; there were 12,295 individuals prior to application of exclusion criteria

^a^∑OH-PAHs include all eight urinary hydroxylated PAH metabolites from four parent compounds (naphthalene, fluorene, phenanthrene, pyrene)

^b^Smoking status defined as current active smoker based on questionnaire, or serum cotinine concentrations >10 ng/mL, defined at ETS exposure

^c^Poverty status is calculated as the ratio of the family’s self-reported income to the family’s poverty threshold
