# Supplementary material for: Urinary polycyclic aromatic hydrocarbon metabolites and mortality in the United States: A prospective analysis
Source: PLoS One. 2021 Jun 4;16(6):e0252719. doi: 10.1371/journal.pone.0252719 (PMC8177506; doi:10.1371/journal.pone.0252719)
Supplement: S2 Table — (DOCX) [file pone.0252719.s005.docx]

S2 Table. Pearson correlation coefficients of eight urinary OH-PAHs (ng/L) from participants participating in NHANES 2001-2014 (N=9739).

|  | Naphthalene | | Fluorene | | Phenanthrene | | Pyrene |
| --- | --- | --- | --- | --- | --- | --- | --- |
|  | 1-NAP | 2-NAP | 3-FLUO | 2-FLUO | 1-PHEN | 2&3-PHEN | 1-PYR |
| 1-NAP | 1.00 |  |  |  |  |  |  |
| 2-NAP | 0.60 | 1.00 |  |  |  |  |  |
| 3-FLUO | 0.68 | 0.71 | 1.00 |  |  |  |  |
| 2-FLUO | 0.68 | 0.72 | 0.95 | 1.00 |  |  |  |
| 1-PHEN | 0.54 | 0.56 | 0.72 | 0.77 | 1.00 |  |  |
| 2&3-PHEN | 0.58 | 0.62 | 0.81 | 0.85 | 0.88 | 1.00 |  |
| 1-PYR | 0.51 | 0.61 | 0.73 | 0.73 | 0.71 | 0.74 | 1.00 |

Abbreviations: 1-NAP = 1-hydroxynaphthalene, 2-NAP = 2-hydroxynaphthalene, 2-FLUO = 2-hydroxyfluorene, 3-FLUO = 3-hydroxyfluorene, 1-PHEN = 1-hydroxyphenanthrene, 2&3-PHEN = sum of 2-hydroxyphenanthrene and 3-hydroxyphenanthrene, 1-PYR = 1-hydroxypyrene
