# Supplementary material for: Urinary polycyclic aromatic hydrocarbon metabolites and mortality in the United States: A prospective analysis
Source: PLoS One. 2021 Jun 4;16(6):e0252719. doi: 10.1371/journal.pone.0252719 (PMC8177506; doi:10.1371/journal.pone.0252719)
Supplement: S3 Table — (DOCX) [file pone.0252719.s006.docx]

S3 Table. Continuous final models^a^ of ∑OH-PAHs and all-cause and cause-specific mortality, excluding participants who died within one and two years of baseline.

|  |  | Exclude deaths within 1 year of baseline | |  | Exclude deaths within 2 years of baseline | |
| --- | --- | --- | --- | --- | --- | --- |
|  |  | Cases | HR_adj_ (95% CI) |  | Cases | HR_adj_ (95% CI) |
| All-cause mortality | Continuous (log_10_ nmol/L) | 860 | 1.38 (1.21, 1.58) |  | 767 | 1.39 (1.19, 1.61) |
|  | Quartile 1 | 226 | Ref |  | 210 | Ref |
|  | Quartile 4 | 248 | 1.68 (1.32, 2.13) |  | 217 | 1.63 (1.29, 2.05) |
| Cancer-specific mortality | Continuous (log_10_ nmol/L) | 151 | 1.19 (0.81, 1.74) |  | 131 | 1.13 (0.73, 1.75) |
|  | Quartile 1 | 34 | Ref |  | 30 | Ref |
|  | Quartile 4 | 46 | 1.59 (0.81, 3.11) |  | 36 | 1.48 (0.70, 3.15) |
| CVD -specific mortality | Continuous (log_10_ nmol/L) | 97 | 1.50 (0.97, 2.31) |  | 91 | 1.57 (1.02, 2.41) |
|  | Quartile 1 | 29 | Ref |  | 27 | Ref |
|  | Quartile 4 | 27 | 2.12 (0.80, 5.61) |  | 26 | 2.26 (0.82, 6.19) |

Abbreviations: CVD = cardiovascular disease

^a^Models adjusted for age (years), gender (male/female), race/ethnicity (non-Hispanic white, non-Hispanic black, Hispanic, other race/ethnicity), smoking status (current, not-current), BMI (kg/m^2^), survey cycle (cycles 1-7), educational attainment (<high school, high school graduate, some college or above), family poverty status (above, at or below family poverty threshold), and urinary creatinine (g/L)
