# Supplementary material for: Urinary polycyclic aromatic hydrocarbon metabolites and mortality in the United States: A prospective analysis
Source: PLoS One. 2021 Jun 4;16(6):e0252719. doi: 10.1371/journal.pone.0252719 (PMC8177506; doi:10.1371/journal.pone.0252719)
Supplement: S4 Table — (DOCX) [file pone.0252719.s007.docx]

S4 Table. Associations^a^ between creatinine-corrected ∑OH-PAHs (nmol/g Cre) and mortality, including urinary creatinine (g/L) as an additional covariate.

| Mortality type | | Continuous (per log_10_-increase) | Quartile 1 | Quartile 2 | Quartile 3 | Quartile 4 | *p-trend^b^* |
| --- | --- | --- | --- | --- | --- | --- | --- |
| All causes  (N=9739) | Cases | 934 | 225 | 226 | 207 | 276 | <0.001 |
|  | HR (95% CI) | 1.39 (1.21, 1.61) | Ref. | 1.15 (0.90, 1.47) | 1.14 (0.90, 1.45) | 1.58 (1.30, 1.92) |  |
| Cancer-specific (N=8862) | Cases | 159 | 41 | 33 | 35 | 50 |  |
|  | HR (95% CI) | 1.15 (0.79, 1.69) | Ref. | 0.96 (0.57, 1.60) | 0.87 (0.49, 1.58) | 1.35 (0.79, 2.30) | 0.30 |
| CVD-specific (N=8975) | Cases | 108 | 24 | 26 | 22 | 36 |  |
|  | HR (95% CI) | 1.49 (0.94, 2.33) | Ref. | 1.24 (0.69, 2.23) | 1.21 (0.52, 2.83) | 1.84 (0.95, 3.57) | 0.10 |

Abbreviations: CVD = cardiovascular disease

^a^Models adjusted for age (years), gender (male/female), race/ethnicity (non-Hispanic white, non-Hispanic black, Hispanic, other race/ethnicity), smoking status (current, not-current), BMI (kg/m^2^), survey cycle (cycles 1-7), educational attainment (<high school, high school graduate, some college or above), family poverty status (above, at or below family poverty threshold), and urinary creatinine (g/L)

^b^Computed using a 'continuous' exposure created out of medians of each quartile of creatinine-corrected log_10_ ΣPAHs
