# Supplementary material for: Urinary polycyclic aromatic hydrocarbon metabolites and mortality in the United States: A prospective analysis
Source: PLoS One. 2021 Jun 4;16(6):e0252719. doi: 10.1371/journal.pone.0252719 (PMC8177506; doi:10.1371/journal.pone.0252719)
Supplement: S5 Table — (DOCX) [file pone.0252719.s008.docx]

S5 Table. Continuous final models^a^ of ΣOH-PAHs and all-cause and cause-specific mortality, not excluding self-reported history of cancer or CVD at baseline.

| Mortality type | | Continuous (per log_10_-increase) | Quartile 1 | Quartile 2 | Quartile 3 | Quartile 4 | *p-trend^b^* |
| --- | --- | --- | --- | --- | --- | --- | --- |
| All causes (N=9739) | Cases | 934 | 240 | 233 | 195 | 266 |  |
|  | ΣOH-PAHs (nmol/L), median | 1.72 | 1.17 | 1.56 | 1.89 | 2.40 |  |
|  | HR_adj_ (95% CI) | 1.39 (1.21, 1.61) | Ref. | 1.16 (0.89, 1.51) | 1.17 (0.89, 1.54) | 1.66 (1.32, 2.09) | <0.001 |
| Cancer-specific (N=9739) | Cases | 222 | 42 | 60 | 52 | 68 |  |
|  | ΣOH-PAHs (nmol/L), median | 1.72 | 1.17 | 1.56 | 1.89 | 2.40 |  |
|  | HR_adj_ (95% CI) | 1.27 (0.91, 1.77) | Ref. | 1.08 (0.69, 1.69) | 1.28 (0.77, 2.14) | 1.48 (0.82, 2.66) | 0.16 |
| CVD-specific (N=9739) | Cases | 173 | 56 | 40 | 35 | 42 |  |
|  | ΣOH-PAHs (nmol/L), median | 1.72 | 1.17 | 1.56 | 1.89 | 2.40 |  |
|  | HR_adj_ (95% CI) | 1.27 (0.89, 1.81) | Ref. | 1.11 (0.57, 2.17) | 1.05 (0.54, 2.05) | 1.38 (0.72, 2.66) | 0.37 |

Abbreviations: CVD = cardiovascular disease

^a^Models adjusted for age (years), gender (male/female), race/ethnicity (non-Hispanic white, non-Hispanic black, Hispanic, other race/ethnicity), smoking status (current, not-current), BMI (kg/m^2^), survey cycle (cycles 1-7), educational attainment (<high school, high school graduate, some college or above), family poverty status (above, at or below family poverty threshold), and urinary creatinine (g/L)

^b^Computed using a 'continuous' exposure created out of medians of each quartile of creatinine-corrected log_10_ ΣPAHs
