# Supplementary material for: Urinary polycyclic aromatic hydrocarbon metabolites and mortality in the United States: A prospective analysis
Source: PLoS One. 2021 Jun 4;16(6):e0252719. doi: 10.1371/journal.pone.0252719 (PMC8177506; doi:10.1371/journal.pone.0252719)
Supplement: S6 Table — (DOCX) [file pone.0252719.s009.docx]

S6 Table. Mutually-adjusted models^a^ examining the association between individual OH-PAHs and all-cause, cancer-specific, and CVD-specific mortality.

|  | All-cause mortality  (N cases=934) | Cancer-specific mortality  (N cases=159) | CVD-specific mortality  (N cases=108) |
| --- | --- | --- | --- |
| Multipollutant models^b^ (nmol/L) | HR_adj_ (95% CI) | HR_adj_ (95% CI) | HR_adj_ (95% CI) |
| Log_10_ 1-hydroxynaphthalene | 1.34 (1.15, 1.57) | 0.91 (0.58, 1.43) | 1.22 (0.81, 1.84) |
| Log_10_ 2- hydroxynaphthalene | 1.00 (0.80, 1.25) | 1.19 (0.72, 1.98) | 1.45 (0.79, 2.66) |
| Log_10_ 2-hydroxyfluorene | 2.13 (1.27, 3.57) | 1.79 (0.67, 4.79) | 3.86 (1.15, 12.94) |
| Log_10_ 3-hydroxyfluorene | 0.66 (0.41, 1.06) | 1.39 (0.54, 3.58) | 0.53 (0.16, 1.75) |
| Log_10_ 1-hydroxyphenanthrene | 0.57 (0.38, 0.85) | 0.66 (0.32, 1.34) | 0.34 (0.13, 0.88) |
| Log_10_ Σ(2- and 3-hydroxyphenanthrene) | 1.40 (1.01, 1.95) | 0.65 (0.30, 1.42) | 2.59 (1.13, 5.90) |
| Log_10_ 1-hydroxypyrene | 0.82 (0.58, 1.15) | 1.19 (0.58, 2.44) | 0.34 (0.12, 0.91) |

Abbreviations: PAH = polycyclic aromatic hydrocarbons, CVD = cardiovascular disease

^a^Models adjusted for age (years), gender (male/female), race/ethnicity (non-Hispanic white, non-Hispanic black, Hispanic, other race/ethnicity), smoking status (current, not-current), BMI (kg/m^2^), survey cycle (cycles 1-7), educational attainment (<high school, high school graduate, some college or above), family poverty status (above, at or below family poverty threshold), urinary creatinine (g/L) in addition to mutual adjustment for OH-PAHs
